# Breast asymmetry: when “left” is not “right”

**DOI:** 10.11604/pamj.2018.29.165.12398

**Published:** 2018-03-20

**Authors:** Nikolaos Garmpis, Christos Damaskos

**Affiliations:** 1 Second Department of Propedeutic Surgery, “Laiko” General Hospital, Medical School, National and Kapodistrian University of Athens, Athens, Greece; 2 Laboratory of Experimental Surgery and Surgical Research NS Christeas, Medical School, National and Kapodistrian University of Athens, Athens, Greece

**Keywords:** Breast, asymmetry, size

## Image in medicine

Intrinsic and extrinsic factors such as hormones may result in asymmetry between double organs such as breast. Breast asymmetries are divided into three types: 1. Volume asymmetry is seen in development disorders of the breast such as macromastia or hypoplasia. This type of breast asymmetry is possibly associated with breast cancer; 2. Shape asymmetry is associated with Poland syndrome and tubular breast; 3. Position asymmetry is usual in patients with scoliosis. There is a variety of surgical techniques in order to cure tis entity. We present a 21-year-old female patient that was referred to our department complaining for breast volume asymmetry. Her past medical history was free of diseases and she didn't take any drugs. Under general anesthesia the patient underwent implantation of a unilateral expander in the hypoplastic left breast and mastopexy in the tuberous right breast. In few months she will proceed in the placement of permanent implant.

**Figure 1:**
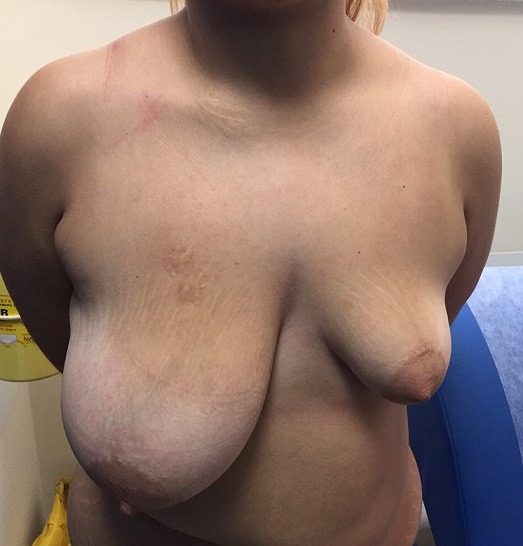
Preoperative view of a 21 year-old female with a severe breast volume asymmetry characterized by a tuberous right breast and a hypoplastic left breast

